# Age features and reference intervals for the concentrations of some essential and toxic elements in laying hens

**DOI:** 10.14202/vetworld.2022.943-952

**Published:** 2022-04-15

**Authors:** Svyatoslav Lebedev, Oleg Zavyalov, and Aleksey Frolov

**Affiliations:** Federal Research Centre of Biological Systems and Agrotechnologies of the Russian Academy of Sciences, Orenburg, Russia

**Keywords:** age, essential and toxic elements, feathers, laying hens, reference intervals

## Abstract

**Background and Aim:**

Micronutrient imbalances pose a severe threat to the health and productivity of livestock and poultry. In this regard, a further stage in feeding science development will control and optimize the intake of mineral substances, including determining the elemental composition in some biosubstrates. One of these biosubstrates can be a feather. However, the amount of available information on the content of trace elements in laying hens is limited, complicating the laboratory data interpretation. Therefore, this study established reference intervals for the concentrations of the main essential and toxic elements in laying hens in different periods of ontogenesis.

**Materials and Methods:**

The study was conducted on clinically healthy Hisex Brown laying hens at the age of 10 (n=150), 30 (n=150), 120 (n=150), 150 (n=150), and 210 (n=150) days. All examined birds were born and raised on the territory of the South Ural biogeochemical province of Russia. The sampling of feathers was carried out by plucking the flight feathers of the wing. Inductively coupled plasma dynamic reaction cell mass spectrometry determined the elemental composition of the feather according to 25 parameters.

**Results:**

The results showed that at the minimum age (10 days), the highest concentrations of chemical elements were observed in laying hens. Subsequently, as they grew older, in the period from the 30^th^ to the 120^th^ day, there was a significant decrease in these indicators. Later, from the 120^th^ to the 150^th^ day, a statistically significant increase in concentrations was replaced. Little growth and relative concentration stability were observed in the last part of the experiment (150-210 days). Chemical element concentrations in feathers were computed in reference ranges for each age group.

**Conclusion:**

The calculated ranges of chemical element concentrations in laying hens can be used to compile norms for their content in the body; however, it is worth noting that these ranges can vary depending on the biogeochemical province of breeding and the bird’s age.

## Introduction

One of the main prerequisites for normal body functioning is the stability of its mineral composition. Mineral substances (cobalt [Co], copper [Cu], iron [Fe], iodine [I], manganese [Mn], Molybdenum [Mo], selenium [Se], zinc [Zn], etc.) are necessary for the normal functioning of almost all biochemical processes in the body. They are part of numerous enzymes, coordinate many biological processes and affect the health and productivity of farm animals. Optimal nutrition with a sufficient level of trace elements assures adequate functioning of the body. Structural, physiological, catalytic, and mechanisms are the most critical [1-5]. Imbalances of trace elements pose a severe threat to the health and productive qualities of farm animals [6] and poultry [7-10], not only in terms of vital elements but also in toxic ones [11]. Toxic metals entering the bodies of animals and poultry in excess amounts can weaken the immune system. It causes oxidative stress [12] negatively affects biochemical parameters, reproductive function, productivity, increases the frequency of heart disease, and leads to the manifestation of various mutations and neoplasms [13-17]. As the science of poultry feeding develops, it becomes evident that controlling and optimizing the intake of mineral substances using noninvasive methods for assessing metabolism in the body, including determining the elemental composition in individual substrates, will be a further stage in the development. Previous studies conducted on beef [18], and dairy cattle [19], cattle breeding, and sports horse breeding [20] confirmed this. The most common substrate for determining the primary conditions of poultry is blood [21,22]. However, the feather is preferred when defining an organism’s elemental status over a lengthy period with the lowest harmful effects on the organism [23-28]. In addition, determining heavy metal levels in bird feathers has also helped monitor the heavy metal contamination of territories [29,30]. Earlier published studies found that the levels of trace elements in feathers largely reflect the exchange pool of the main essential and toxic elements in broilers and laying hens [31,32]. Simultaneously, the amount of available information on the content of microelements in the feather of birds, particularly laying hens, is limited at different periods of ontogenesis. Moreover, there are no reference intervals calculated using the recommendations of the American Society for Veterinary Clinical Pathology Quality Assurance and Laboratory Standard Guidelines [33], which complicates the objective interpretation of laboratory data.

The study aimed to assess age characteristics and establish reference intervals for the concentrations of the main essential and toxic elements of laying hens bred in a separate biogeochemical province using inductively coupled plasma dynamic reaction cell mass spectrometry (ICP-DRC-MS) methods. The intervals specified in this study will allow timely detection of elementosis in laying hens at different stages of ontogenesis and prescribing the appropriate mineral complexes for the metabolic correction of the elemental status.

## Materials and Methods

### Ethical approval

The Local Ethics Committee of the Orenburg State University, Orenburg, Russia, approved the protocol of the present investigation (No. 2021/68 dated February 15, 2021). All animal studies were performed following the ethical standards laid down in the 1964 Declaration of Helsinki and its later amendments.

### Study period and location

The study was conducted in March and April 2021 in the conditions of three poultry farms (Orenburgskaya Poultry Farm CJSC, Sputnik OJSC, Uralsky Broiler JSC) located on the territory of Orenburg Region, Russia. 

### Environmental conditions of the experimental region

The territory of the Southern Urals is located in the depths of the Eurasian continent, far from the oceans. This continentality significantly affects the climate, soils, and vegetation. The climate is sharply continental; hot summers are accompanied by dry winds and winters are cold with stable snow cover. There is a large absolute amplitude of air temperature fluctuations, reaching 85°C. In summer, heated air enters the region from Central Asia and Kazakhstan, so the air temperature reaches 40-43°C. In the cold season, frosts intensify due to the cold air from Siberia, and the temperature drops to −40°C. Precipitation averages 374 mm/year.

### Experimental design

The territory of the Orenburg region belongs to the regions with favorable ecological conditions. The studies were conducted on clinically healthy Hisex Brown laying hens at the age of ten (n=150), 30 (n=150), 120 (n=150), 150 (n=150), and 210 (n=150) days. The keeping conditions for all examined chickens were identical. Birds were kept in metal cages of 145 cm (width)×70 cm (depth)×250 cm (height) with mesh floors. The cells were arranged in five tiers on either side of the feeding passage. The air temperature in the workshop for rearing laying hens was 15-18°C, humidity-60-70%. Air exchange per 1 kg of live weight of adult laying hens in the cold period of the year was 0.7 m^3^/h, in the warm period-5 m^3^/h. Incandescent lamps with a power of 40-75 W are used to illuminate poultry houses. The illumination in the area of the feeders was 10-15 lux. The length of daylight hours in the poultry house for the adult population of laying hens was 14 h, in the rooms for young animals 7.5 h. Laying hens were vaccinated against Newcastle, Marek, Gumboro disease, infectious bronchitis chickens, egg drop syndrome, pneumovirus, reovirus tenosynovitis, salmonellosis, and coccidiosis.

In general, the nutrient content in the diet of the experimental birds met the needs of laying hens during the periods of starter, growth, and laying [34]. The complete feed given to laying hens in the period of 0-8 weeks included wheat (40%), corn (20%), soybean meal (24%), sunflower meal (7%), meat flour (1.23%), and oil sunflower (3.64%); in the period 9-15 weeks: wheat (40%), barley (34%), granulated bran (7%), extruded soybeans (1.3%), sunflower meal (12%), meat flour (1.3%), sunflower oil (1.5%); in the period 16-49 weeks: Wheat (56%), granulated bran (10%), soybean meal (4%), sunflower meal (16%), meat meal (2.2%), and sunflower oil (1.5%). Furthermore, the bird was fed a vitamin-mineral premix (microelements-calcium [Ca], phosphorous [P], sodium [Na], potassium [K], chlorine [Cl], macroelements-Fe, Cu, Zn, Mn, J, Se, Vitamins A D3, E, K3, B1–B6, B12, Bc, and H). Furthermore, the birds were free to water through automatic nipple drinkers. The content of chemical elements in the daily diet given to laying hens in the 10 days preceding sampling is presented in Table-1.

**Table 1:** Content of chemical elements in native substance of diet of laying hens at different age periods.

Element	Age, days				

	10	30	120	150	210
Macroelements (mg/kg)					
Ca	135636	282575	942880	1296460	1743120
K	104208	217100	342880	471460	549600
Mg	23856	49700	137120	188540	224160
Na	12288	25600	96720	132990	88560
P	63552	132400	354080	486860	490320
Essential elements (mg/kg)					
Co	3.10	6.45	16.1	23.3	24.24
Cr	37.2	77.5	207	284	423
Cu	184	384	991	1362	1496
Fe	2472	5150	16560	22770	21480
I	10.55	22.0	24.7	34.0	91.0
Mn	1872	3900	13680	18810	15240
Se	7.15	14.9	19.2	26.4	24.5
Zn	1197	2495	7135	9810	9877
Conditionally essential elements (mg/kg)					
B	154	321	348	479	774
Li	0.938	1.95	5.19	7.12	7.43
Ni	54.4	113	165	227	324
Si	857	1786	3722	5118	6439
V	6.85	14.3	62.8	86.3	132
Toxic elements (mg/kg)					
As	0.345	0.720	2.15	2.96	10.4
Al	201	419	1166	1603	1626
Sr	201	420	1147	1577	1579
Pb	1.21	2.52	2.89	3.98	5.39
Sn	0.0852	0.177	0.136	0.187	0.360
Cd	0.833	1.73	5.10	7.02	9.72
Hg	0.0362	0.0754	0.232	0.319	0.216

As=Arsenic, Al=Aluminum, Sr=Strontium, Pb=Lead, Sn=Tin, Cd=Cadmium, Hg=Mercury, B=Boron, Li=Lithium, Ni=Nickel, Si=Silicon, V=Vanadium, Co=Cobalt, Cr=Chromium, Cu=Copper, Fe=Iron, I=Iodine, Mn=Manganese, Se=Selenium, Zn=Zinc, Ca=Calcium, K=Potassium, Mg=Magnesium, Na=Sodium, P=Phosphorus

### Sample collection

Feathers were sampled by taking the flight feathers of the wing. In addition, the proximal part of the feather, weighing at least 0.4 g, was collected for research. Feather samples were taken from three heads from 50 randomly selected cages located in different workshop sections on the territory of each poultry farm under study.

### Elemental analysis

The obtained feather samples were washed in acetone (Sigma-Aldrich Co., USA) to remove external contamination and then rinsed thrice in deionized water (18 MW cm). After washing, the samples were dried at 60°C to a stable weight. Fifty milligrams of feather samples were placed into Teflon tubes containing 5 mL of concentrated nitric acid (Sigma-Aldrich Co.) and subsequently digested in the microwave system (Bergh of Products + Instruments GmbH, Eningen, Germany). Bergh of SW-4 DAP-40 microwave system. The obtained solutions were poured into 15-mL polypropylene test tubes, adjusted to the final volume of 15 mL with deionized water, and thoroughly mixed by shaking in the closed test tubes. Essential, conditionally essential (Ca, K, Magnesium [Mg], Na, P, Co, chromium [Cr], Cu, Fe, I, Li, Mn, Se, Si, Strontium [Sr], vanadium [V], Zn), toxic, and potentially toxic (arsenic [As], boron [B], cadmium [Cd], mercury [Hg], nickel [Ni], lead [pb], and Stannum [Sn]) trace elements in the samples were assessed using a NexION 300D spectrometer (PerkinElmer, USA) equipped with an ESI SC-2 DX4 autosampler (Elemental Scientific Inc., Omaha, NE, USA). Standard solutions with various concentrations of trace elements prepared from Universal Data Acquisition Standards Kits (PerkinElmer Inc.) are used to calibrate the system. The 10 g/L Yttrium and Rhodium Pure Single-Element Standard (PerkinElmer Inc.) used for internal online standardization. Permanent analysis of the certified reference material of hair GBW09101 (Shanghai Institute of Nuclear Research, Shanghai, China) was employed for laboratory quality control. A study was conducted before and after every round of examination. The recovery rate for all trace elements analyzed was between 88 and 107%. The Center for Biotic Medicine laboratory (Moscow, Russia) is an IUPAC company associate and participates in the Occupational and Environmental Medicine External Quality Assessment Schemes system.

### Statistical analysis

The significance of differences was tested using the Mann–Whitney U test. The significance level (p) was considered at ≤0.05. The data were processed using the Statistica 10.0 software package (Stat Soft Inc., USA). The reference intervals were estimated using the American Society for Veterinary Clinical Pathology Quality Assurance and Laboratory Standard Guidelines [33]. Briefly, after percentile two-sided exclusion of outliers, the robust method was applied to assess reference intervals and 90% confidence intervals (90% CI) for the lower and upper limits. The assessment of the reference intervals was performed using Reference Value Advisor for MS Excel (Microsoft Inc., USA).

## Results

A comparative analysis of the data obtained revealed a significant difference in the main essential and toxic elements in different periods of ontogenesis (Table-2).

**Table 2:** The concentration of main essential and toxic elements in laying hens in different periods of ontogenesis.

Element	Age, days				

	10	30	120	150	210
Macroelements (mg/kg)					
Ca	2009±746	1241±1113^a^	671±436^ab^	1590±1841^c^	1132±850^a^
K	2298±633	1900±810	560±365^ab^	601±329^ab^	733±553^ab^
Mg	359±83.1	234±148^a^	173±103^ab^	229±106^ac^	296±169^b^
Na	1731±481	1645±853	748±391^ab^	876±387^ab^	959±278^ab^
P	1559±569	982±408^a^	316±277^ab^	372±322^ab^	281±156^ab^
Essential elements (mg/kg)					
Co	0.150±0.107	0.126±0.168	0.0382±0.0363^ab^	0.0992±0.0823^c^	0.0764±0.0682^ab^
Cr	1.93±0.509	3.52±2.30^a^	2.12±0.899^b^	3.84±1.99^ac^	2.61±1.86^b^
Cu	8.34±3.32	13.1±17.8	6.92±3.62^b^	11.8±7.99^c^	10.1±3.20
Fe	110±30.2	134±193	35.5±25.6^ab^	93.1±79.0^c^	69.4±59.7^ab^
I	7.52±4.33	1.34±1.86^a^	0.311±0.334^ab^	0.417±0.434^ab^	0.694±1.15^ab^
Mn	17.5±7.33	8.52±12.9^a^	4.71±7.91^a^	5.82±8.76^a^	8.69±10.8^a^
Se	0.791±0.215	0.630±0.192^a^	0.368±0.114^ab^	0.450±0.137^abc^	0.491±0.085^ab^
Zn	246±47.0	318±400	193±257	191±145^b^	186±82.2^ab^
Conditionally essential elements (mg/kg)					
B	2.07±0.499	1.75±1.20	0.709±0.563^ab^	0.948±0.755^ab^	1.25±1.19^ab^
Li	0.0522±0.0194	0.0393±0.0362	0.0134±0.0133^ab^	0.0202±0.0141^abc^	0.0300±0.0243^a^
Ni	0.715±0.227	2.71±4.02	1.08±2.53^b^	8.43±12.1^abc^	1.91±4.03^d^
Si	24.9±17.14	44.7±25.9a	42.1±18.4^a^	53.0±28.9^ac^	21.4±20.4^bd^
V	0.299±0.187	0.236±0.190	0.139±0.128^ab^	0.223±0.222^c^	0.217±0.201
Toxic elements (mg/kg)					
As	0.0473±0.0124	0.0343±0.0252^a^	0.0210±0.0101^ab^	0.0323±0.0164^ac^	0.0385±0.0212
Al	33.4±9.90	18.0±26.5^a^	6.76±6.08^ab^	6.82±5.30^ab^	11.7±13.3^ad^
Sr	3.31±1.28	1.74±1.80^a^	0.882±0.962^ab^	1.71±1.49^ac^	1.63±1.64^a^
Pb	1.18±0.581	1.79±3.09	0.933±2.14	0.675±1.01^b^	1.00±1.44
Sn	0.199±0.0964	14.0±28.2	6.81±23.7	18.0±7.3^abc^	4.30±13.8^bd^
Cd	0.0232±0.0100	0.201±0.383	0.0693±0.206^b^	0.673±0.923^abc^	0.135±0.385^d^
Hg	0.0141±0.0140	0.0074±0.0073^a^	0.0352±0.0225^ab^	0.0334±0.0213^ab^	0.0481±0.0302^ab^

a=p≤0.05 comparing 10 days; b=p≤0.05 comparing 30 days; с=p≤0.05 comparing 120 days; d=p ≤ 0.05 comparing 150 days. As=Arsenic, Al=Aluminum, Sr=Strontium, Pb=Lead, Sn=Tin, Cd=Cadmium, Hg=Mercury, B=Boron, Li=Lithium, Ni=Nickel, Si=Silicon, V=Vanadium, Co=Cobalt, Cr=Chromium, Cu=Copper, Fe=Iron, I=Iodine, Mn=Manganese, Se=Selenium, Zn=Zinc, Ca=Calcium, K=Potassium, Mg=Magnesium, Na=Sodium, P=Phosphorus

As a result, the overwhelming list of chemical elements was discovered to have the highest concentrations in 10-day laying hens. The highest concentrations of the overwhelming list of chemical elements were found in laying hens at 10 days of age. Furthermore, compared to the 30 days of age, there was a considerable drop in Ca (p≤0.01), Mg (p≤0.01), P (p≤0.001), I (p≤0.001); Mn (p≤0.01), Se (p≤0.01), As (p≤0.05), Al (p≤0.01), Sr (p≤0.01), and Hg (p≤0.01) as they grew older.

At the age of 120 days, this difference increased and was, respectively, for Ca – 66.6% (p≤0.001); K – 75.6% (p≤0.001); Mg – 51.8% (p≤0.001); Na – 56.7% (p≤0.001); P – 79.7% (p≤0.001); Co – 74.7% (p≤0.001); I – 95.9% (p≤0.001); Mn – 73.1% (p≤0.001); Se – 53.5% (p≤0.001); B – 65.7% (p≤0.001); Li – 75.0% (p≤0.001); V – 53.5% (p≤0.001); As – 55.3% (p≤0.001); Al – 79.8% (p≤0.001); and Sr – 73.4% (p≤0.001). Si concentration in feathers of birds of 30 and 120 days of age was higher - by 76.5 (p≤0.01) and 68.9% (p≤0.01); Cr – by 82.4% (p≤0.01) and 9.8%.

We recorded the minimum concentrations of Ca, K, Mg, Na, P, Co, Cr, Cu, Fe, I, Mn, Se, B, Li, Ni, V, As, Al, Sr, Cd, and Hg for the entire study period of egg production at the age of 120 days. The exception was Si, the concentration of which did not confirm the general pattern and was higher relative to the same indicator calculated for the age of 150 days by 25.8% (p≤0.001). In general, for the period from 120^th^ to 210^th^ day, the fact of a gradual increase was observed in the concentrations of Ca (p≤0.01), Mg (p≤0.001), Na (p≤0.01), Co (p≤0.001), Cu (p≤0.001), Fe (p≤0.001); I (p≤0.05), Mn (p≤0.05), Se (p≤0.001), B (p≤0.01), Li (p≤0.001), V (p≤0.05), As (p≤0.001), Al (p≤0.05), Sr (p≤0.01), and Hg (p≤0.05). Simultaneously, the concentration levels of Co (p≤0.001), Cr (p≤0.001), Cu (p≤0.001), Fe (p≤0.001), Ni (p≤0.001), Si (p≤0.05), V (p≤0.05), Sr (p≤0.01), Sn (p≤0.001), and Cd (p≤0.001) increased in laying hens in the period from 120^th^ to 150^th^ day, after which they statistically insignificantly decreased to 210-day age.

Because the average values of the concentrations of chemical elements in laying hens in different periods of ontogenesis (10, 30, and 120 days) had significantly significant differences, the reference intervals were calculated separately for each listed period (Tables-3-5). Consequent to the absence of a significant difference in the chemical composition of feathers at the age of 150 and 210 days, the reference intervals were calculated together (Table-6).

**Table 3:** Reference ranges of concentrations of essential and toxic elements in laying hens at the age of 10-20 days.

Element	2.5 (90% CI)	97.5 (90% CI)
Macroelements (mg/kg)		
Ca	847 (709-984)	3116 (2609-3623)
K	1102 (968-1235)	3510 (3086-3934)
Mg	188.0 (169-207)	542 (487.1-596.9)
Na	752.0 (660-843)	2478 (2176-2780)
P	583.0 (489-676)	2265 (1903-2627)
Essential elements (mg/kg)		
Co	0.0682 (0.0474-0.0901)	0.508 (0.349-0.667)
Cr	0.545 (0.482-0.608)	2.73 (2.41-3.05)
Cu	3.09 (2.55-3.63)	15.8 (13.1-18.6)
Fe	50.0 (44.0-56.0)	182 (160-203.8)
I	3.07 (2.29-3.85)	16.2 (12.1-20.3)
Mn	9.84 (8.04-11.6)	30.2 (24.7-35.8)
Se	0.357 (0.314-0.400)	1.16 (1.02-1.30)
Zn	161 (147-174)	346 (317-374)
Conditionally essential elements (mg/kg)		
B	1.32 (1.18-1.46)	3.09 (2.76-3.42)
Li	0.0283 (0.0244-0.0335)	0.085 (0.0713-0.0982)
Ni	0.328 (0.282-0.374)	1.12 (0.963-1.28)
Si	0.545 (0.381-0.709)	67.6 (47.2-87.9)
V	0.0894 (0.0653-0.114)	0.751 (0.545-0.957)
Toxic elements (mg/kg)		
As	0.0203 (0.0184-0.0231)	0.0723 (0.0644-0.0803)
Al	17.5 (15.2-19.8)	52.5 (45.7-59.3)
Sr	1.47 (1.22-1.72)	5.29 (4.40-6.18)
Pb	0.574 (0.451-0.697)	2.63 (2.06-3.20)
Sn	0.0624 (0.0491-0.0753)	0.423 (0.333-0.513)
Cd	0.0085 (0.0074-0.0105)	0.0395 (0.0324-0.0461)
Hg	0.00183 (0.0010-0.0026)	0.0523 (0.0292-0.0754)

As=Arsenic, Al=Aluminum, Sr=Strontium, Pb=Lead, Sn=Tin, Cd=Cadmium, Hg=Mercury, B=Boron, Li=Lithium, Ni=Nickel, Si=Silicon, V=Vanadium, Co=Cobalt, Cr=Chromium, Cu=Copper, Fe=Iron, I=Iodine, Mn=Manganese, Se=Selenium, Zn=Zinc, Ca=Calcium, K=Potassium, Mg=Magnesium, Na=Sodium, P=Phosphorus

**Table 4:** Reference ranges of concentrations of essential and toxic elements in laying hens at the age of 20-40 days.

Element	2.5 (90% CI)	97.5 (90% CI)
Macroelements (mg/kg)		
Ca	236 (201-271)	5226 (4448-6004)
K	623 (579-667)	3381 (3142-3620)
Mg	94.3 (84.4-104)	757 (677-836)
Na	494 (451-536)	3279 (2997-3561)
P	290 (270-310)	1812 (1687-1937)
Essential elements (mg/kg)		
Co	0.0124 (0.0091-0.0142)	0.746 (0.581-0.911)
Cr	0.398 (0.355-0.441)	10.5 (9.40-11.7)
Cu	3.77 (2.92-4.62)	93.5 (72.4-114)
Fe	14.9 (11.4-18.5)	939 (714-1163)
I	0.111 (0.0853-0.137)	6.87 (5.29-8.45)
Mn	0.506 (0.379-0.633)	63.2 (47.3-79.1)
Se	0.239 (0.227-0.251)	1.18 (1.12-1.24)
Zn	113 (89.4-136)	1918 (1518-2318)
Conditionally essential elements (mg/kg)		
B	0.356 (0.315-0.397)	4.99 (4.42-5.56)
Li	0.0113 (0.0094-0.0132)	0.188 (0.160-0.216)
Ni	0.122 (0.0924-0.152)	15.0 (11.3-18.7)
Si	0.508 (0.459-0.557)	104 (94.0-114)
V	0.0322 (0.0274-0.0362)	0.878 (0.761-0.995)
Toxic elements (mg/kg)		
As	0.0103 (0.0084-0.0115)	0.115 (0.101-0.129)
Al	2.13 (1.61-2.65)	137 (103-170)
Sr	0.327 (0.271-0.383)	7.82 (6.48-9.16)
Pb	0.0963 (0.0682-0.124)	12.7 (9.09-16.4)
Sn	0.0444 (0.0301-0.0593)	92.5 (61.7-123)
Cd	0.0062 (0.0043-0.0074)	1.43 (0.912-1.84)
Hg	0.0018 (0.0015-0.0021)	0.0204 (0.0172-0.0236)

As=Arsenic, Al=Aluminum, Sr=Strontium, Pb=Lead, Sn=Tin, Cd=Cadmium, Hg=Mercury, B=Boron, Li=Lithium, Ni=Nickel, Si=Silicon, V=Vanadium, Co=Cobalt, Cr=Chromium, Cu=Copper, Fe=Iron, I=Iodine, Mn=Manganese, Se=Selenium, Zn=Zinc, Ca=Calcium, K=Potassium, Mg=Magnesium, Na=Sodium, P=Phosphorus

**Table 5:** Reference ranges of concentrations of essential and toxic elements in laying hens at the age of 100-140 days.

Element	2.5 (90% CI)	97.5 (90% CI)
Macroelements (mg/kg)		
Ca	101 (85.1-116)	2049 (1726-2372)
K	73.1 (61.6-84.7)	1421 (1197-1645)
Mg	31.6 (27.1-36.2)	514 (439-588)
Na	165 (144-185)	1712 (1495-1929)
P	33.1 (26.1-40.2)	1143 (899-1386)
Essential elements (mg/kg)		
Co	0.00132 (0.0010-0.0016)	0.135 (0.104-0.166)
Cr	0.213 (0.191-0.235)	4.36 (3.91-4.81)
Cu	1.08 (0.940-1.22)	13.9 (12.2-15.7)
Fe	2.41 (1.99-2.83)	98 (80.9-115)
I	0.0284 (0.0213-0.0352)	1.19 (0.884-1.50)
Mn	0.136 (0.0813-0.191)	33.5 (19.9-47.2)
Se	0.0972 (0.0903-0.104)	0.599 (0.554-0.644)
Zn	36.8 (25.0-48.7)	1268 (859-1676)
Conditionally essential elements (mg/kg)		
B	0.124 (0.0973-0.143)	2.38 (1.92-2.84)
Li	0.0004 (0.0003-0.0005)	0.0523 (0.0395-0.0654)
Ni	0.0193 (0.0085-0.0294)	9.52 (4.14-14.9)
Si	3.81 (3.41-4.21)	82.7 (73.9-91.5)
V	0.020 (0.016-0.025)	0.460 (0.357-0.563)
Toxic elements (mg/kg)		
As	0.0023 (0.0024-0.0033)	0.0475 (0.0423-0.0521)
Al	1.26 (0.986-1.53)	22.7 (17.7-27.6)
Sr	0.0534 (0.0393-0.0675)	3.94 (2.90-4.98)
Pb	0.0223 (0.0101-0.0353)	9.76 (4.33-15.2)
Sn	0.0112 (0.0023-0.0204)	79.6 (12.4-146)
Cd	0.0002 (0.0001-0.0004)	0.766 (0.211-1.32)
Hg	0.0047 (0.0040-0.0054)	0.0753 (0.0642-0.0863)

As=Arsenic, Al=Aluminum, Sr=Strontium, Pb=Lead, Sn=Tin, Cd=Cadmium, Hg=Mercury, B=Boron, Li=Lithium, Ni=Nickel, Si=Silicon, V=Vanadium, Co=Cobalt, Cr=Chromium, Cu=Copper, Fe=Iron, I=Iodine, Mn=Manganese, Se=Selenium, Zn=Zinc, Ca=Calcium, K=Potassium, Mg=Magnesium, Na=Sodium, P=Phosphorus

**Table 6:** Reference ranges of concentrations of essential and toxic elements in laying hens at the age of 140-230 days.

Element	2.5 (90% CI)	97.5 (90% CI)
Macroelements (mg/kg)		
Ca	353 (288-417)	2769 (2265-3273)
K	187 (152-221)	2069 (1691-2447)
Mg	122 (105-138)	669 (576-761)
Na	550 (511-588)	1655 (1539-1771)
P	107 (92.5-121)	571 (494-648)
Essential elements (mg/kg)		
Co	0.0022 (0.0017-0.0027)	0.201 (0.157-0.245)
Cr	0.344 (0.285-0.403)	6.37 (5.27-7.47)
Cu	5.1 (4.71-5.49)	14.1 (13.0-15.1)
Fe	7.21 (5.71-8.71)	168 (133-203)
I	0.0853 (0.0514-0.119)	4.68 (2.80-6.56)
Mn	0.130 (0.0913-0.169)	28.71 (20.05-37.37)
Se	0.337 (0.323-0.351)	0.649 (0.622-0.676)
Zn	115 (102-127)	352 (314-389)
Conditionally essential elements (mg/kg)		
B	0.0734 (0.0563-0.0905)	3.65 (2.81-4.49)
Li	0.0037 (0.0030-0.0044)	0.0749 (0.0603-0.0895)
Ni	0.0325 (0.0153-0.0481)	14.87 (7.28-22.46)
Si	3.56 (2.74-4.38)	68.4 (52.6-84.2)
V	0.0133 (0.0104-0.0165)	0.634 (0.492-0.776)
Toxic elements (mg/kg)		
Al	0.566 (0.410-0.722)	40.8 (29.6-52.1)
As	0.0165 (0.0143-0.0185)	0.0801 (0.0693-0.0902)
Sr	0.166 (0.126-0.206)	4.44 (3.36-5.52)
Pb	0.0194 (0.0123-0.0262)	4.47 (2.90-6.04)
Sn	0.0395 (0.0093-0.0702)	51.6 (11.2-91.9)
Cd	0.0002 (0.0001-0.0004)	1.39 (0.427-2.35)
Hg	0.0083 (0.0072-0.0094)	0.0965 (0.0812-0.111)

As=Arsenic, Al=Aluminum, Sr=Strontium, Pb=Lead, Sn=Tin, Cd=Cadmium, Hg=Mercury, B=Boron, Li=Lithium, Ni=Nickel, Si=Silicon, V=Vanadium, Co=Cobalt, Cr=Chromium, Cu=Copper, Fe=Iron, I=Iodine, Mn=Manganese, Se=Selenium, Zn=Zinc, Ca=Calcium, K=Potassium, Mg=Magnesium, Na=Sodium, P=Phosphorus

Significant differences in the magnitude of the percentile intervals were discovered when the borders of the generated intervals were compared in the analyzed age periods. For example, at the age of 30 days, the most outstanding 97.5 percentile value was found for Ca, Mg, Na, Co, Cr, Cu, Fe, Mn, Se, Zn, B, Li, NI, Si, As, Al, Sr, Pb, Sn, and Cd, and the lowest values for K, P, I was observed at the age of 10 days. However, lying hens had minimum values of 2.5 percentile for all examined elements at 120 days of age, except for Si, which had minimum values at 10 days.

## Discussion

It can be stated that the highest concentrations of chemical elements were observed in laying hens at the age (of 10 days). Subsequently, as they grew older, in the period from the 30^th^ to the 120^th^ day, there was a significant decrease in these elements, which was later replaced by a statistically significant increase in concentrations in the period from the 120^th^ to the 150^th^ day. Minimal growth and relative stability of concentrations characterize the experiment’s final period (150-210 days).

It was assumed that changes in the concentrations of chemical elements in the bird’s feathers would depend on the level of the latter’s intake of feed, as we previously showed for horses [20]. However, despite the trend of an increase in the concentration of chemical elements in the diet with an increase in the age of the bird in our study (Table-1), the dynamics of the concentration of mineral substances in feathers, in contrast, had a general tendency to decrease (Table-2). The most indicative in this regard is a sharp decrease in the concentration of chemical elements in the feathers of the experimental birds, against the background of an increase in their daily intake with a diet, which was noted in the period from 10 to 30 days. The previous studies indicated that age could significantly affect the metabolism of trace elements in the body of poultry [35,36]. In particular, it was shown that adult chickens were characterized by substantially higher Pb and Cd accumulation levels in meat and liver than chickens [37,38]. In our experiment, CD level in laying hens in different ontogenic periods increased by 2-29 times concerning 10 days of age. An explanation of this fact is possible, considering the earlier studies. A significant age-related increase in the concentrations of toxic elements in various biological substrates of poultry is associated with a more extended intake of toxic metals from feed and drinking water [39,40].

However, this hypothesis was not confirmed in a comparative analysis of the results for the concentrations of As, Al, Sr, and Pb, the content of which at an early age (10 and 30 days) was significantly higher than in later periods. These differences may be associated with the metabolism of metallothionein, a protein synthesized in the body of animals in response to the intake of heavy metals from the external environment [41]. Sex hormones control the amount of metallothionein in the body [42], the concentration of which depends on age [43].

The significant fact is that the minimum concentrations of almost all the evaluated chemical elements were observed at the end of molting at 120 days. Accompanying a natural decrease in feed intake and inhibition of the metabolism of trace elements in the body of laying hens [44]. Elements such as Ca, phosphorus (P), Zn, Mn, and Cu, and others play an essential role as cofactors of enzyme systems associated with the process of bone mineralization and the quality of eggshells in laying hens [45,46]. In this regard, the noted decrease in the concentration of trace elements in poultry at the age of 120 days may also be associated with the depletion of their body reserves due to the onset of the egg production period and the initiation of the eggshell mineralization process, including due to the labile reserve in the modular bone [47].

The data obtained in our experiment compared with the data on the content of trace elements in birds raised in different regions were to understand the influence of the breeding biogeochemical province on the elemental status of laying hens. Comparative analysis showed that the data on the Zn content obtained in our experiment were higher than the results obtained for chickens raised in Malaysia [48] and Korea [49] and are practically comparable with those obtained in Pakistan [50]. The results on the concentration of Cu and Mn in our study were similar to the study conducted in Korea [49,51] and, at the same time, were significantly lower than the study conducted in Belgium [52]. The received data on the content of Pb and Cd in laying hens bred in the biogeochemical province of the South Urals (Russia) were significantly lower than those observed in South Korea [49,53], Pakistan [50], Belgium [52],China [54], the Republic of Kosovo [55], and and were practically similar to the results obtained for Nigeria [56], as concentrations were lower than the data obtained for Belgium [57] and Malaysia [48].

The observed difference in the content of toxic metals in feathers may be related to background pollution with toxic metals in the surveyed areas [55,58,59]. Simultaneously, the methodology for the selection and assessment of the mineral composition of substrates can have a significant impact on the results obtained, which makes it challenging to work on a comparative analysis of the results obtained in various studies [60,61].

## Conclusion

Thus, the concentrations of most chemical elements in feather-laying hens varied significantly depending on age. In this regard, the age factor must be considered when developing reference intervals for specific regions. In this study, reference concentration intervals were determined for 25 chemical elements (Al, As, B, Ca, Cd, Co, Cr, Cu, Fe, I, K, Li, Mg, Mn, Na, Ni, P, Pb, Se, Si, Sn, Hg, Sr, V, and Zn) in the feathers of physiologically healthy laying hens of the Hisex Brown cross at different periods of ontogenesis (10-20; 20-40; 100-140; and 140-230 days). The established reference intervals are proposed to be used to detect elementosis in laying hens bred in the South Ural biogeochemical province.

## Authors’ Contributions

SL: Carried out general management and developed experimental methods. OZ: Did statistical analysis and drafted the manuscript. AF conducted experiments, analyzed, and interpreted data. All authors read and approved the final manuscript.
